# Gene expression in lungs of mice lacking the 5-hydroxytryptamine transporter gene

**DOI:** 10.1186/1471-2466-9-19

**Published:** 2009-05-10

**Authors:** Daniel Crona, Julie Harral, Serge Adnot, Saadia Eddahibi, James West

**Affiliations:** 1 Division of Pulmonary Sciences and Critical Care Medicine, University of Colorado Health Sciences Center, Denver, Colorado, USA; 2 INSERM U841 and Département de Physiologie Explorations Fonctionnelles, Hôpital H. Mondor, AP-HP, Créteil, France; 3 Division of Allergy, Pulmonary and Critical Care Medicine, Vanderbilt University, Nashville, Tennessee, USA

## Abstract

**Background:**

While modulation of the serotonin transporter (5HTT) has shown to be a risk factor for pulmonary arterial hypertension for almost 40 years, there is a lack of in vivo data about the broad molecular effects of pulmonary inhibition of 5HTT. Previous studies have suggested effects on inflammation, proliferation, and vasoconstriction. The goal of this study was to determine which of these were supported by alterations in gene expression in serotonin transporter knockout mice.

**Methods:**

Eight week old normoxic mice with a 5-HTT knock-out (5HTT-/-) and their heterozygote(5HTT+/-) or wild-type(5HTT+/+) littermates had right ventricular systolic pressure(RVSP) assessed, lungs collected for RNA, pooled, and used in duplicate in Affymetrix array analysis. Representative genes were confirmed by quantitative RT-PCR and western blot.

**Results:**

RVSP was normal in all groups. Only 124 genes were reliably changed between 5HTT-/- and 5HTT+/+ mice. More than half of these were either involved in inflammatory response or muscle function and organization; in addition, some matrix, heme oxygenase, developmental, and energy metabolism genes showed altered expression. Quantitative RT-PCR for examples from each major group confirmed changes seen by array, with an intermediate level in 5HTT +/- mice.

**Conclusion:**

These results for the first time show the in vivo effects of 5HTT knockout in lungs, and show that many of the downstream mechanisms suggested by cell culture and ex vivo experiments are also operational in vivo. This suggests that the effect of 5HTT on pulmonary vascular function arises from its impact on several systems, including vasoreactivity, proliferation, and immune function.

## Background

Idiopathic pulmonary arterial hypertension (IPAH) is a disease characterized by pulmonary vasoconstriction, inflammation, and vascular remodeling, whose early events and molecular etiology are still obscure. Defects in serotonin (5-HT) signaling have long been associated with idiopathic pulmonary arterial hypertension. From 1965 to 1972, the first appetite depressant-induced epidemic of pulmonary hypertension occurred in Europe following the release of aminorex[1]. During the 1990's, French researchers reported increased incidence of pulmonary hypertension among a patient population that was administered derivatives of the medication fenfluramine[2]. Dexfenfluramine, which is the active enantiomer of fenfluramine and used to treat obesity in patients, was considered to be the chief culprit behind the increase in cases of pulmonary hypertension[2,3]. Dexfenfluramine acts as a substrate for the serotonin transporter (5-HTT), causing increased extracellular 5-HT by a mechanism involving exchange of drug molecules for intracellular 5-HT[4]. Dexfenfluramine also causes overexpression of 5-HTT, but with the net effect of increased available 5-HT[3,5].

Attenuation of hypoxic PAH in experimental mice can occur pharmacologically through the administration of dexfenfluramine[3]. Since increased 5-HT potentiates the development of PAH in rodents[6] and likely in humans[7], the simplest explanation for these data is through direct effect on signaling through 5-HTT, rather than by impact on 5-HT itself. Mice with a specific gene knock-out for 5-HTT (5-HTT^-/-^) are also somewhat protected against hypoxic pulmonary hypertension[8,9], with slightly lower right ventricular systolic pressure (RVSP) in response to chronic hypoxia.

Transgenic mice overexpressing 5-HTT in smooth muscle[10] or under its own promoter[9] spontaneously develop PAH, although the mechanism is still unclear. While 5-HTT is overexpressed in hypoxia, the rate of 5-HT uptake has been reported as reduced.

There are several plausible mechanisms by which the loss of 5HTT could protect against hypoxic PAH. Serotonin is a mitogen, and can act as a smooth muscle mitogen through 5-HTT[11]. Loss of 5-HTT might thus protect against PASMC proliferation. Another potential mechanism lies in the well-characterized impact of 5-HT on smooth muscle tone, through receptor signaling[12,13] and inhibition of K-channels[14]. 5HTT^-/- ^mice have substantially lowered circulating 5-HT[8], and by analogy to 5HTT^-/- ^rats likely plasma 5-HT[15], which should result in decreased 5-HT-derived tone and thus protection against IPAH. Finally, 5-HT modulates cytokine networks in the lung[16], including in a feedback loop with interleukin-1β and p38 MAPK[17].

The 5-HT pathway thus impacts several systems with relevance to IPAH, including proliferation, tone, and inflammation. The goal of this study was to examine changes in gene expression in 5-HTT-/- mouse lung, to determine *in vivo *which of the potential pathways was the likely molecular source of their protection against PAH. We conducted the study in normoxic mice, which lack phenotype, in order to isolate the effects of the serotonin transporter knockout in the absence of confounding factors caused by hypoxia or increased pulmonary pressures.

## Methods

### Animals

5HTT-/- mice were constructed as previously described[8]. Wild-type, 5-HTT heterozygote or knockout mice were allowed to reach adulthood at ambient pressure in Denver, Colorado (85 KPa; ambient pressure at sea level is 100 KPa). The breeding colony was maintained as 5HTT^+/- ^(heterozygote), so all mice used were littermates. Mice were genotyped by polymerase chain reaction using primers specific to the wild-type allele (ATATCCAATGGGTACTCCGCAG and TGGTGAATCTCAGCCACCAG) and the neomycin resistance gene used in the knock-out (TCGACGTTGTCACTGAAGCG and GGATACTTTCTCGGCAGGAGC).

Eight week old mice were anesthetized with ketamine 200 mg/kg and xylazine 10 mg/kg, the right jugular surgically exposed. Right ventricular (RV) pressure was measured using a 1.4 French Pressure Volume Conductance System SPR-839 (Millar Instruments, Houston, Texas), which was inserted into the RV through the surgically exposed right jugular vein. The hemodynamic phenotyping results were continuously recorded using a Millar MPVS-300 unit coupled to a Powerlab 8-SP A/D converter, acquired at 1000 Hz. All hemodynamic phenotyping results were captured to a Macintosh G4 computer utilizing Chart5.3 software. Heparin was injected into the circulation prior to sacrifice, the left atria removed, lungs flushed through the right ventricle, and lungs collected and flash frozen in liquid nitrogen. All animal studies were approved by the UCHSC intramural animal care and use committee.

### Affymetrix arrays

All RNA for the gene arrays consisted of sex-matched pooled samples from three different animals, to reduce variability, with two Mouse Genome 430 2.0 arrays used for each condition (a total of six arrays from 18 pooled animals are thus reported). Samples were prepared for Affymetrix arrays using 2.5 μg of total lung RNA according to the Affymetrix GeneChip Expression Analysis Manual. All array results have been submitted to the NCBI gene expression and hybridization array data repository (GEO, ), as series GSE11035.

Affymetrix cel files were imported into dChip [18], normalized to median overall signal intensity, and the perfect match/mismatch model used to determine per-gene hybridization strength. Pairwise comparisons between 5HTT wildtype and knockout mouse arrays were used to determine changed genes, with same-direction changes in all four pairwise comparisons needed for a gene to be considered changed. Gene ontology was determined using the Classify Genes tool within dChip[18], with gene ontology files downloaded from the Gene Ontology Consortium [19].

Analysis focused on gene ontology groups rather than highly changed individual genes for several reasons. First, different strains can use different genes to accomplish the same phenotypic end[20]; pathways are thus more important than individual genes. Next, arrays are actually rather poor at determining quantitative changes, as shown by results in this study. Finally, our goal was to determine overall pathways altered by loss of 5HTT, rather than individual genes; selection of a low required fold change while maintaining a low false-discovery rate is optimal for this process.

### Western blots

Western blots were performed as previously described[21], using Klf4 rabbit polyclonal (1:500 dilution, CeMines, Golden, CO) and Kcne4 mouse monoclonal (1:300 dilution, Jackson ImmunoResearch Laboratories, West Grove PA). Equal protein loading was determined by Bradford assay; blots were stripped and reprobed with antibodies to β-actin to confirm this. A single strong band at the appropriate size was assumed to indicate specificity.

### Quantitative RT-PCR

Primers were designed using sequences downloaded from Genbank with the Applied Biosystems ABI *Primer Express *program(AB, Foster City, CA). Each primer was searched against BLAST to ensure that it did not match any known gene aside from that for which it was designed (especially other family members). Upon receipt, each primer was tested and titered to ensure it gave a single, clean band of the appropriate size. Primer sequences are listed in Additional File 1. RNA was made from snap-frozen whole mouse lung using a Qiagen RNeasy mini kit (Valencia, CA), and first strand cDNA was created from 1 μg total RNA using a Qiagen QuantiTect^® ^Reverse Transcription Kit (Valencia, CA). Quantitative RT-PCR was carried out using SYBR Green chemistry in a 7300 Real Time PCR System (Applied Biosystems, Foster City, CA). Each measurement was made in triplicate and expressed relative to the detection of the housekeeping gene hypoxanthine guanine phosphoribosyl transferase (HPRT). Absence of contaminating genomic DNA was confirmed by PCR of no-RT control with cogenomic HPRT primers.

## Results

### 5-HTT^-/- ^mice have normal RVSP

While 5HTT^-/- ^mice had never previously been found to have abnormal RVSP, we wished to verify this before proceeding. Mice at 8 weeks of age were weighed and anesthetized in preparation of closed-chested catheterization. Right ventricular systolic pressure (RSVP) and heart rate for each experimental mouse was obtained by inserting a catheter into the right ventricle through a surgically exposed right jugular vein. Under normoxic conditions, with ambient pressure and spontaneous ventilation, RVSP and heart rate were unchanged between 5-HTT^+/+^, 5-HTT^+/- ^and 5-HTT^-/- ^mice (not shown).

### 5-HTT^-/- ^mouse lungs have alterations in multiple pathways with relevance to PAH

RNA was extracted from homogenized whole lung from 5-HTT^+/+^, 5-HTT^+/- ^and 5-HTT^-/- ^mice, with RNA pooled from 3 samples to reduce individual variability for each chip. Two chips, with pools from different animals, were used for wild-type, heterozygote, and homozygote 5-HTT knockouts; all animals were littermates. RNA was used to probe Affymetrix MOE430 v2 arrays. Affymetrix Cel files were further analyzed with dChip.

Using relatively low stringency for comparisons, including a minimum 1.2× fold change between any pair of samples with a minimum threshold for absolute change between any pair, we found 124 genes changed between from 5-HTT^+/+ ^and 5-HTT^-/- ^mice with a false discovery rate of 7%. Using the same criteria and swapping samples between groups reduced this to 6–10 genes, indicating that a large majority of the 124 genes likely represented genotype-dependent changes. These were sorted into gene ontology groups by dChip.

**Figure 1:**
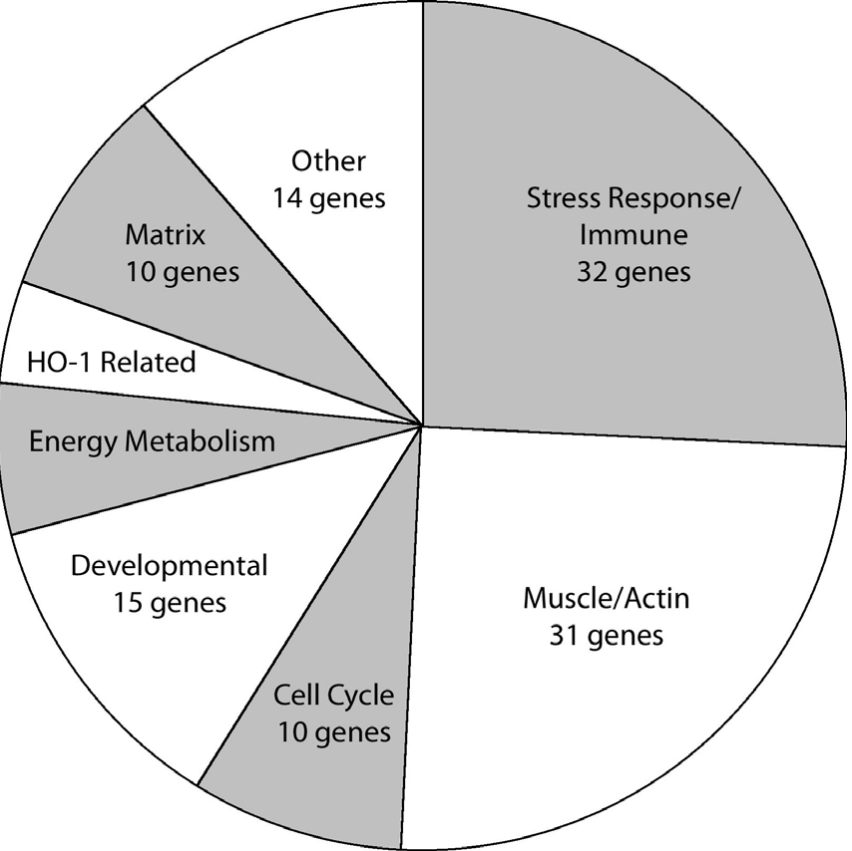
**Distribution of ontology groups of 124 genes differentially regulated in 5HTT^-/- ^mouse whole lung as compared to littermate controls**.

Half of these genes fell into one of two broad gene ontology groups; stress response and immune related genes, and genes related to muscle structure, vasoreactivity, or actin dynamics (Figure 1, Additional File 2). Other categories included cell cycle and apoptosis, developmental, energy metabolism, heme-oxygenes (HO-1), and matrix related genes (Additional file 3). Where a gene fell into more than one ontology group, for the purpose of these tables it was placed in the group for which references seemed to predominate. Changes in 5-HTT^+/- ^mice by array were similar to those in 5-HTT^-/- ^mice (Additional files 2, 3). Absolute changes shown are in arbitrary units of hybridization intensity.

### Quantitative RT-PCR shows dose effect and greater changes than arrays

**Figure 2:**
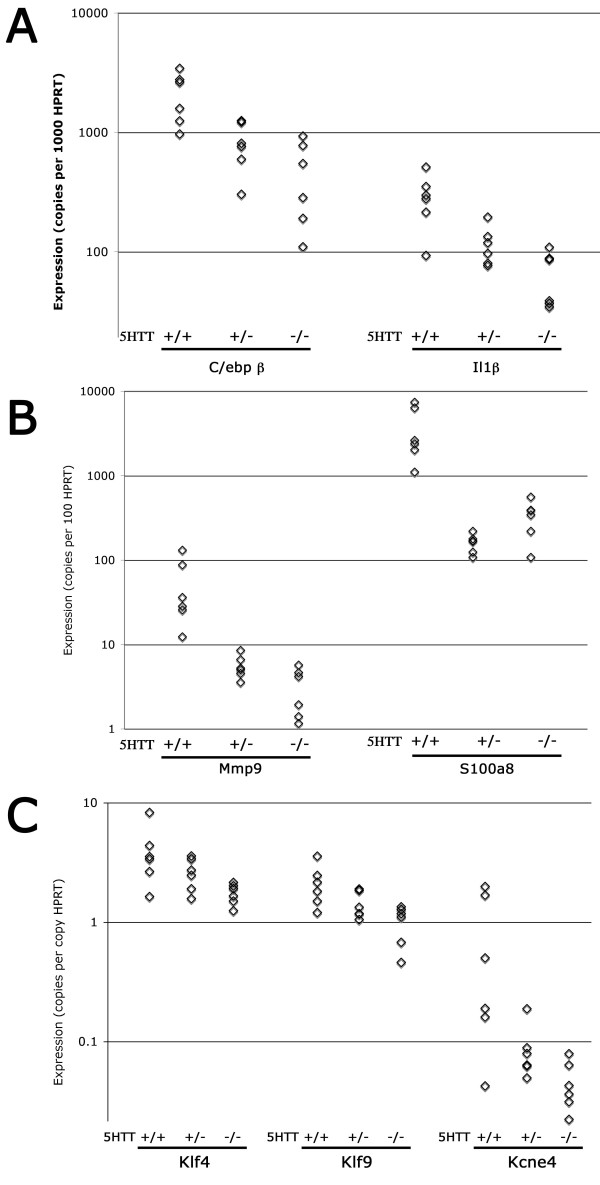
**Quantitative RT-PCR confirmation of array changes for selected genes**. Each point corresponds to RNA from an individual mouse. All changes are significant by ANOVA as p < .01 except Klf4 and Kcne4 which are significant at p < .05. Expression in 5HTT^-/- ^is significantly different than expression in 5HTT^+/+ ^for all genes at p < .05 by Tukey's Post Hoc.

To confirm results obtained by Affymetrix arrays, selected genes from different categories were analyzed by quantitative RT-PCR against cDNA derived from six individual mice per group. These included inflammation-related genes C/ebpβ and Il1β(Figure 2A), matrix related genes Mmp9 and S100a8 (Figure 2B), muscle related genes Klf4 and Kcne4, and the developmental gene Klf9 (Figure 2C). We found in each case that quantitative RT-PCR confirmed the change seen on the Affymetrix arrays, with two important differences (Additional file 4 and 5). First, in most cases the fold change was much greater by quantitative RT-PCR than by array, and second, there was an evident dose effect in six of seven genes by quantitative RT-PCR which was not apparent by array.

### Western blot confirms decreased protein expression of Klf4 and Kcne4

**Figure 3:**
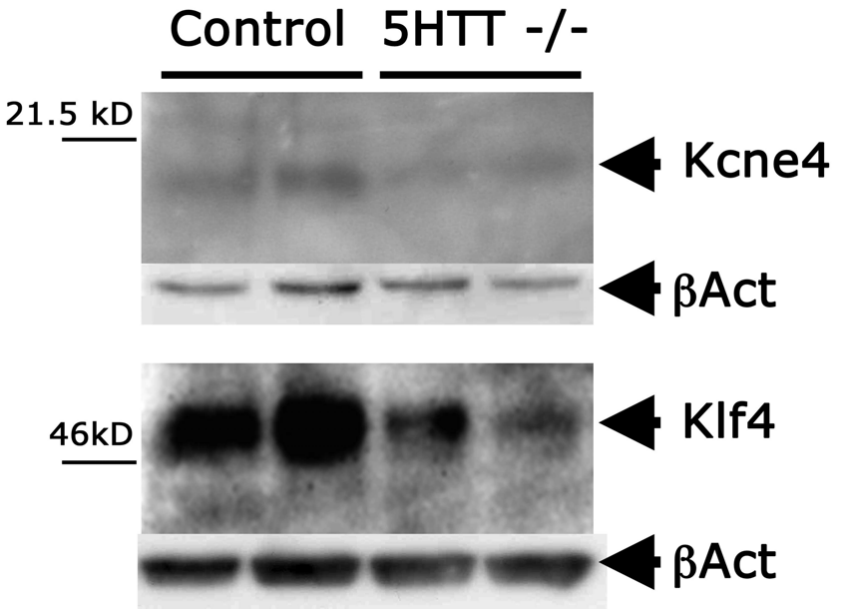
**Western blot analysis shows decreased protein expression of Klf4 and Kcne4 in protein lysates from whole lungs of 5HTT^-/- ^mice compared to controls; βactin demonstrates even protein loading, and was present at approximately 45 kD**.

While most of the quantitative PCR results had very strong statistical values, scatter in values was greater and the corresponding statistical significance weaker for muscle developmental gene Klf4 and the voltage gated potassium channel Kcne4. Western blot analysis was therefore performed to confirm changes in protein level of these genes, with comparison to β-actin for protein loading. We found even protein loading with decreased Klf4 and Kcne4 protein across samples (Figure 3), with average decreases in protein levels of 3.5× and 2.0× for Klf4 and Kcne4 respectively by densitometry.

## Discussion

There has been a long association between serotonin signaling and pulmonary arterial hypertension[1], with many mechanisms proposed for its effect, including impact on vascular tone[12,13], proliferation, and inflammation[16]. The goal of this study was to determine which of these mechanisms was plausible in vivo by examining the gene expression consequences in homogenized whole lung of serotonin transporter (5HTT) knockout and heterozygosity as compared to wild-type mice. We found that pathways related to all of the proposed mechanisms are altered in 5-HTT knockout mice. This is the first report of broad in vivo consequences in lung of loss of the serotonin transporter. We found a relatively limited number of genes consistently changed, including stress response, actin organization, cell cycle, energy metabolism, heme oxygenase, matrix, and developmental genes (Fig 1, Additional files 2, 3). Representative genes from major groups were assayed by quantitative RT-PCR, confirming their change, and showing intermediate levels of change in 5HTT heterozygote mice (Fig 2). Genes with weak p-values by quantitative RT-PCR were also confirmed by densitometry and western blot (Fig 3).

We were surprised that by gene array 5HTT heterozygote mice seemed closer to the 5HTT knockouts than to the wild-type mice, as it appears that haploinsufficiency in 5HTT does not alter 5 HT uptake, at least in brain[22]. However, the simplest interpretation of this is that alterations in gene expression are the result of 5HTT function directly, rather than secondary to 5 HT level. There are multiple examples in the literature showing a dose-effect, in which 5HTT heterozygotes have a phenotype midway between knockouts and wild-type mice, in MDMA response[22], platelet aggregation[23], and serotonin receptor density in areas of the brain[24].

Expression analysis on brains of 5HTT knockout mice has recently been performed[25]. Hippocampus with 5HTT knockout also shows relatively few differentially regulated genes (only 30), but with only one gene in common, S100A9. There are a few additional genes with related function: for example, in brain period homolog 2 (Per2) has altered expression, while in lung it is Per1. However, for the most part the gene lists do not overlap either in pathway or in specific gene. This likely indicates very different signaling consequences of loss of 5HTT in brain and lung.

The classes of genes discovered as dysregulated are particularly interesting because they are similar to classes of genes found dysregulated in two mouse models which develop PAH, including mice expressing a BMPR2 mutation and mice with a vasoactive intestinal peptide (VIP) knockout. BMPR2 is the gene mutated in most familial PAH; adult mice expressing smooth-muscle specific mutations in BMPR2 develop PAH, although with a phenotype somewhat dependent on the mutation used[26,27]. VIP knockout mice develop moderate PAH, including smooth muscle proliferation and perivascular inflammation[28,29].

5HTT^-/- ^mice, which are protected against hypoxic PAH, show broad decreases in immune response genes (Additional file 2), while both VIP^-/- ^and BMPR2 mutant mice show increases in immune response gene expression[26,28,30]. Both VIP^-/- ^and BMPR2 mutant mice show increases in collagen, and BMPR2 mice show increased matrix metalloproteinase(MMP) and matrix-related gene S100A8; 5HTT-/- mice have decreased MMP and S100A8. 5HTT-/- mice have cell cycle changes that tend towards lower proliferation, such as an increase in the cell cycle inhibitor Fabp3 and a decrease in oncogene Fosl2(Additional file 3); VIP^-/- ^and BMPR2 mutant mice have changes trending towards an increase in proliferation. 5HTT^-/- ^mice have changes in vasoreactivity related genes which suggest increased tone, including increases in muscle structural genes and decreases in potassium channels (Additional file 2). This is similar to although less pronounced than trends seen in both VIP^-/- ^and BMPR2 mutant mice; one would not expect this to be protective.

## Conclusion

The goal of this study was to examine changes in gene expression in 5-HTT-/- mouse lung, to determine *in vivo *which of the potential pathways was the likely molecular source of their protection against PAH. We found that all of the potential pathways, including proliferation, inflammation, and vasoconstriction, among others, were altered. However, of these only alterations in proliferation and inflammation seem likely to underlie a protective effect of loss of 5-HTT.

## Competing interests

The authors declare that they have no competing interests.

## Authors' contributions

DC carried out the quantitative RT-PCR and western blot experiments and helped to draft the manuscript. JH phenotyped mice and prepared RNA for array experiments. SA developed the mouse model and conceived of the study. SE developed the mouse model and provided animals for the study. JW analyzed the microarray results, drafted the manuscript, and coordinated the study.

## Pre-publication history

The pre-publication history for this paper can be accessed here:



## Supplementary Material

Additional File 1 **Table of primer sets used for quantitative RT-PCR in Figure **2. Forward and reverse primers, with product lengths, used for quantitative RT-PCR for targets in Figure 2. Click here for file

Additional File 2 **Table of gene array results**. Table of stress response and smooth muscle-related genes differentially expressed in lungs of serotonin knockout and heterozygote mice. Click here for file

Additional File 3 **Table of gene array results**. Table of cell cycle, developmental, energy metabolism, heme oxygenase related, matrix, and other genes differentially expressed in lungs of serotonin knockout and heterozygote mice. Click here for file

Additional File 4 **Comparison of fold change found by quantitative RT-PCR to that found by array for selected genes.** Click here for file

Additional File 5 **Numeric quantitative RT-PCR results**. Raw quantitative RT-PCR data used to construct figure 2, additional file 4. Click here for file
