# Supplementary material for: Gene expression in lungs of mice lacking the 5-hydroxytryptamine transporter gene
Source: BMC Pulm Med. 2009 May 10;9:19. doi: 10.1186/1471-2466-9-19 (PMC2688484; doi:10.1186/1471-2466-9-19)
Supplement: Additional File 1 — Table of primer sets used for quantitative RT-PCR in Figure 2. Forward and reverse primers, with product lengths, used for quantitative RT-PCR for targets in Figure 2. [file 1471-2466-9-19-S1.pdf]

Table 1: Primer Sequences

Gene	Forward	Reverse	Product
Cebbp	AAGAGCCGCGACAAGGC	GGCAGCTGCTTGAACAAGTTC	146
Il1b	CGTGGACCTTCCAGGATGAG	AATGGGAACGTCACACACCAG	119
Kcne4	AGCAGTGGTAATGGCAATGAATAC	CATGTAGCCCAGCATGATTCC	90
Klf4	GGCTGTGGGTGGAAATTCG	CGCACTTCTGGCACTGAAAG	91
Klf9	CCTCCCATCTTAAAGCCCATTAC	TCATCCGAGCGCGAGAAC	97
Mmp9	CAGGAGTCTGGATAAGTTGGGTCTAG	ACGCCCCTTGCTGAACAG	100
S100A8	CCATGCCCTCTACAAGAATG	ATCACCATCGCAAGGAACTC	150
