# Supplementary material for: Gene expression in lungs of mice lacking the 5-hydroxytryptamine transporter gene
Source: BMC Pulm Med. 2009 May 10;9:19. doi: 10.1186/1471-2466-9-19 (PMC2688484; doi:10.1186/1471-2466-9-19)
Supplement: Additional File 2 — Table of gene array results. Table of stress response and smooth muscle-related genes differentially expressed in lungs of serotonin knockout and heterozygote mice. [file 1471-2466-9-19-S2.pdf]

Table 2
Fold Change: average fold increase or decrease compared to average of wild-type arrays
Absolute Change: Wild-type array values substracted from homozygote knockout array values

Symbol	gene	EntrezGene	Fold Change +/-	Fold Change -/-	Absolute Change
**Stress response or immune-related genes**					
Atf3	activating transcription factor 3	11910	-1.8	-2.4	-150
Atf4	activating transcription factor 4	11911	-1.2	-1.3	-230
Cebpb	CCAAT/enhancer binding protein, beta	12608	-1.5	-1.5	-480
Cebpbd	CCAAT/enhancer binding protein, delta	12609	-1.7	-2.2	-970
Chka	choline kinase alpha	12660	-1.2	-1.4	-160
Csf3r	colony stimulating factor 3 receptor	12986	-3.2	-2.2	-100
Dusp1	dual specificity phosphatase 1	19252	-1.5	-1.6	-1280
Gadd45g	growth arrest and DNA-damage 45 gamma	23882	-1.8	-2.3	-160
Il1b	interleukin 1 beta	16176	-3.4	-3.3	-220
Il8rb	interleukin 8 receptor, beta	12765	-6.0	-4.7	-160
Klf15	Kruppel-like factor 15	66277	-1.5	-1.6	-240
Klf2	Kruppel-like factor 2 (lung)	16598	-1.1	-1.2	-400
Klf3	Kruppel-like factor 3 (basic)	16599	-1.2	-1.3	-170
Lpin2	lipin 2	64898	-1.1	-1.3	-140
Mat2a	methionine adenosyltransferase II, alpha	232087	-1.1	-1.3	-290
Myd116	myeloid differentiation gene 116	17872	-1.3	-1.5	-200
Nfkbia	nuclear factor of kappa inhibitor, alpha	18035	-1.5	-1.6	-780
Nr4a1	nuclear receptor subfamily 4, group A, member 1	15370	-1.2	-1.5	-380
Nr4a2	nuclear receptor subfamily 4, group A, member 2	18227	-1.7	-2.1	-130
Plunc	palate, lung, and nasal carcinoma associated	18843	2.1	2.4	2030
Ptpn6	protein tyrosine phosphatase 6	15170	1.3	1.6	230
Rasl11b	RAS-like, family 11, member B	68939	-1.2	-1.3	-160
Slfn4	schlafen 4	20558	-3.7	-3.4	-190
Socs2	suppressor of cytokine signaling 2	216233	-1.2	-1.3	-290
Socs3	suppressor of cytokine signaling 3	12702	-1.5	-1.3	-120
Stfa2l1	stefin A2 like 1	268885	-3.8	-3.7	-100
Stk17b	serine/threonine kinase 17b	98267	-1.4	-1.4	-190
Tiparp	TCDD-inducible poly(ADP-ribose) polymerase	99929	-1.2	-1.4	-150
Tnfaip3	tumor necrosis factor, alpha-induced protein 3	21929	-1.4	-1.5	-110
Tsc22d3	TSC22 domain family 3	14605	-1.3	-1.5	-230
Wasf2	WAS protein family, member 2	242687	-1.2	-1.2	-130
Zfp36	zinc finger protein 36	22695	-2.1	-1.9	-520
**Muscle structure, vasoreactivity, actin dynamics**					
Actc1	actin, alpha, cardiac	11464	1.2	1.4	880
Aldoa	aldolase 1, A isoform	11674	1.0	1.3	230
Arid5b	AT rich interactive domain 5B (Mrf1 like)	71371	-1.3	-1.3	-110
Arl4c	ADP-ribosylation factor-like 7	320982	-1.3	-1.3	-120
Calcrl	calcitonin receptor-like	54598	1.5	1.7	200
Chd3	chromodomain helicase DNA binding protein 3	216848	-1.4	-1.9	-360
Cnn1	calponin 1	12797	1.3	1.4	160
Csrp3	cysteine and glycine-rich protein 3	13009	1.4	1.9	200
Dgat2	diacylglycerol O-acyltransferase 2	67800	1.5	1.6	110
Dgkh	Diacylglycerol kinase, eta	380921	-1.2	-1.3	-180
Edn1	endothelin 1	13614	-1.6	-1.8	-450
HSPB6	heat shock protein, alpha-crystallin-related, B6	243912	1.2	2.1	190
Ifrd2	Interferon-related developmental regulator 1	15982	-1.2	-1.3	-120
Ivns1abp	influenza virus NS1A binding protein	117198	1.0	1.3	190
Kcne4	potassium voltage-gated channel, Isk-related 4	57814	-1.4	-1.7	-90
Kcnj15	potassium inwardly-rectifying channel, J15	16516	-1.3	-1.4	-130
Klf4	Kruppel-like factor 4 (gut)	16600	-1.3	-1.4	-430
Mb	myoglobin	17189	1.9	2.9	570
Myh11	myosin, heavy polypeptide 11, smooth muscle	17880	1.2	1.3	210
Pde4b	phosphodiesterase 4B, cAMP specific	18578	-1.2	-1.3	-260
Pfn1	profilin 1	18643	1.4	2.1	540
Qk	quaking	19317	-1.1	-1.2	-210
RhoB	ras homolog gene family, member B	11852	-1.2	-1.3	-550
Rock2	Rho-associated coiled-coil forming kinase 2	19878	-1.1	-1.2	-150
Sfrs6	splicing factor, arginine/serine-rich 6	67996	1.1	1.2	280
Sln	sarcolipin	66402	1.7	2.3	460
Tcap	titin-cap	21393	1.6	2.0	160
Tpm1	tropomyosin 1, alpha	22003	1.3	1.6	510
Tpm2	tropomyosin 2, beta	22004	1.2	1.4	380
Ttn	Titin	22138	1.5	2.3	540
Unc45b	cardiomyopathy associated 4	217012	-1.3	-1.7	-170
