# Supplementary material for: Gene expression in lungs of mice lacking the 5-hydroxytryptamine transporter gene
Source: BMC Pulm Med. 2009 May 10;9:19. doi: 10.1186/1471-2466-9-19 (PMC2688484; doi:10.1186/1471-2466-9-19)
Supplement: Additional File 3 — Table of gene array results. Table of cell cycle, developmental, energy metabolism, heme oxygenase related, matrix, and other genes differentially expressed in lungs of serotonin knockout and heterozygote mice. [file 1471-2466-9-19-S3.pdf]

Table 3
Fold Change: average fold increase or decrease compared to average of wild-type arrays
Absolute Change: Wild-type array values substracted from homozygote knockout array values

	Symbol	gene	EntrezGene	Fold Change +/-	Fold Change -/-	Absolute Change
**Cell Cycle & Apoptosis**						
	Bcl2l11	BCL2-like 11 (apoptosis facilitator)	12125	-1.0	-1.3	-220
	Ccnl1	cyclin L1	56706	-1.1	-1.2	-310
	Ddit4	DNA-damage-inducible transcript 4	74747	-2.0	-2.4	-690
	Dnajc3b	DnaJ (Hsp40) homolog, subfamily C, member 3	19107	-1.1	-1.2	-170
	Fabp3	fatty acid binding protein 3, muscle and heart	14077	1.6	1.6	120
	Fosl2	fos-like antigen 2	14284	-1.3	-1.6	-260
	Hecw2	HECT, C2 and WW domain containing 2	329152	-1.5	-1.5	-140
	Pim3	proviral integration site 3	223775	-1.7	-2.0	-580
	Sox4	SRY-box containing gene 4	20677	1.2	1.3	290
	Tnfsf10	tumor necrosis factor superfamily, member 10	22035	1.4	1.4	130
**Developmental**						
	Ankrd11	ankyrin repeat domain 11	77087	-1.2	-1.4	-130
	App	Amyloid beta (A4) precursor protein	11820	1.4	1.3	100
	Axud1	AXIN1 up-regulated 1	215418	-2.0	-2.7	-240
	Bmp1	Bone morphogenetic protein 1	12153	-1.8	-1.7	-220
	Btg2	B-cell translocation gene 2, anti-proliferative	12227	-1.4	-1.6	-460
	Fzd4	frizzled homolog 4 (Drosophila)	14366	-1.2	-1.4	-410
	Gdap10	ganglioside-induced differentiation-associated 10	14546	-1.2	-1.3	-160
	Klf7	Kruppel-like factor 7 (ubiquitous)	93691	-1.1	-1.2	-200
	Klf9	Kruppel-like factor 9	16601	-1.3	-1.5	-200
	Nrp1	neuropilin 1	18186	1.8	1.9	340
	Prickle1	prickle like 1	106042	1.2	1.3	190
	Prickle2	Prickle-like 2	243548	1.3	1.3	130
	Rnf144a	Ring finger protein 144	108089	1.4	1.6	120
	Zbtb16	Zinc finger and BTB domain containing 16	235320	-1.0	-1.9	-200
	Zfp503	zinc finger protein 503	218820	1.1	1.3	370
**Energy Metabolism**						
	Cycs	cytochrome c, somatic	13063	1.2	1.2	140
	Gdpd1	glycerophosphodiester phosphodiesterase 1	66569	-1.9	-4.3	-540
	Lpl	lipoprotein lipase	16956	1.3	1.3	400
	Ndufc1	NADH dehydrogenase 1, subcomplex unknown, 1	66377	1.1	1.3	160
	Ndufs8	NADH dehydrogenase Fe-S protein 8	225887	1.2	1.2	180
	Nnt	nicotinamide nucleotide transhydrogenase	18115	1.1	1.3	150
	Slc2a3	solute carrier family 2 member 3	20527	-2.2	-1.8	-130
**Heme Oxygenase Related**						
	Alas1	Aminolevulinic acid synthase 1	11655	-1.4	-1.5	-130
	Bach1	BTB and CNC homology 1	12013	-1.1	-1.4	-210
	Cyp2e1	cytochrome P450, family 2, e1	13106	1.4	1.5	140
	Egln3	EGL nine homolog 3	112407	1.3	1.5	140
	Elk-3	ELK3, member of ETS oncogene family	13713	1.4	1.5	120
**Extracellular Matrix & Cell Adhesion**						
	Adamts15	A disintegrin-like and metalloprotease 15	235130	-1.2	-1.4	-160
	Clec4d	C-type lectin domain family 4, member d	17474	-3.9	-3.7	-140
	Col1a1	procollagen, type I, alpha 1	12842	1.1	1.3	230
	Fn1	fibronectin 1	14268	1.4	1.5	550
	Lgals1	lectin, galactose binding, soluble 1	16852	1.2	1.4	350
	Mmp9	matrix metalloproteinase 9	17395	-2.5	-2.6	-120
	Mt2	metallothionein 2	17750	-1.7	-1.4	-150
	Npnt	nephronectin	114249	-1.0	-1.2	-230
	s100a8	S100 calcium binding protein A8 (calgranulin A)	20201	-5.7	-4.2	-1920
	s100a9	S100 calcium binding protein A9 (calgranulin B)	20202	-6.3	-5.0	-1730
**Other Functions**						
	Ddost	dolichyl-di-phosphooligosaccharide glycotransferase	13200	1.2	1.4	160
	Eif4a1	eukaryotic translation initiation factor 4A1	13681	1.2	1.4	200
	Fbxo39	F-box protein 39	327959	7.4	13.7	760
	Gda	guanine deaminase	14544	-1.5	-1.4	-100
	Grina	glutamate receptor, ionotropic	66168	-1.3	-1.3	-260
	H3f3b	H3 histone, family 3B	15081	-5.5	-4.6	-230
	Lyve1	extra cellular link domain-containing 1	114332	-1.1	-1.3	-280
	Msi2	Musashi homolog 2 (Drosophila)	76626	-1.8	-3.8	-570
	Ndel1	nuclear distribution gene E-like homolog 1	83431	-1.3	-2.7	-180
	Per1	period homolog 1	18626	-1.6	-2.3	-430
	Sgk	serum/glucocorticoid regulated kinase	20393	-1.3	-1.3	-420
	Slc10a6	solute carrier family 10 member 6	75750	-1.2	-1.5	-230
	Vezf1	vascular endothelial zinc finger 1	22344	1.4	1.3	120
	Zswim6	zinc finger, SWIM domain containing 6	67263	-1.1	-1.2	-150
