# Supplementary material for: Gene expression in lungs of mice lacking the 5-hydroxytryptamine transporter gene
Source: BMC Pulm Med. 2009 May 10;9:19. doi: 10.1186/1471-2466-9-19 (PMC2688484; doi:10.1186/1471-2466-9-19)
Supplement: Additional File 4 — Comparison of fold change found by quantitative RT-PCR to that found by array for selected genes. [file 1471-2466-9-19-S4.pdf]

Table 4

	Quant RT-PCR		Array	
	+/-	-/-	+/-	-/-
CebpB	-2.6	-4.4	-1.5	-1.5
Il1b	-2.5	-4.4	-3.4	-3.3
Kcne4	-8.6	-16.6	-1.4	-1.7
Klf4	-1.5	-2.3	-1.3	-1.4
Klf9	-1.5	-2.1	-1.3	-1.5
Mmp9	-9.6	-16.9	-2.5	-2.6
S100a8	-22.7	-10.9	-5.7	-4.2
